# Systematic review and meta-analysis of the impact of abnormal expression of long non coding RNA on the prognosis of acute myeloid leukemia

**DOI:** 10.3389/fgene.2025.1524449

**Published:** 2025-02-04

**Authors:** Guihong Liu, Liangliang Sun, Peng Lv, Rong Qiao, Lihang Wang, Arong Jin

**Affiliations:** ^1^ Graduate School, Inner Mongolia Medical University, Hohhot, China; ^2^ Inner Mongolia Autonomous Region People’s Hospital, Hohhot, China; ^3^ Graduate School, Baotou Medical College, Inner Mongolia University of Science and Technology, Baotou, China

**Keywords:** long noncoding RNA (LncRNA), acute myeloid leukemia (AML), prognosis, meta-analysis, review-systematic

## Abstract

**Objective:**

Long non-coding RNA (lncRNA) is aberrantly expressed in a variety of tumor diseases. To date, its specific role in acute myeloid leukemia (AML) has not been fully elucidated. This study aims to evaluate the association between aberrant lncRNA expression and poor prognosis in AML patients, and to systematically assess the relationship between aberrant lncRNA expression and AML prognosis.

**Methods:**

We conducted a comprehensive literature search in PubMed, Embase, Cochrane Library, CNKI (China National Knowledge Infrastructure), WanFang (China Wanfang Database), VIP (China VIP Database), and Sinomed (China Biomedical Literature Database) to identify relevant Chinese and English articles. The search period covered from the inception of these databases to 4 August 2024. Articles were screened according to predefined inclusion and exclusion criteria, and meta-analysis was performed using Stata.

**Results:**

A total of 25 articles were included in the analysis. Aberrant lncRNA expression was significantly associated with reduced overall survival (univariate *HR* = 2.46, 95%*CI* 2.11–2.88, *P* < 0.001; multivariate *HR* = 2.46, 95%*CI* 2.11–2.88, *P* < 0.001), event-free survival (*HR* = 1.51, 95%*CI* 1.19–1.90, *P* = 0.001), recurrence-free survival (*HR* = 2.82, 95%*CI* 2.03–3.91, *P* < 0.001), and disease-free survival (*HR* = 2.390, 95%*CI* 1.037–5.507, *P* = 0.041). These findings were statistically significant. The 25 articles collectively identified 22 lncRNAs whose aberrant expression was associated with AML prognosis. Notably, multiple studies highlighted the aberrant expression of lncRNA CRNDE, ZEB2-AS1, and TUG1 as being particularly relevant to AML prognosis. Our meta-analysis revealed that high expression of lncRNA CRNDE and TUG1 was associated with reduced overall survival, while high expression of lncRNA ZEB2-AS1 was linked to decreased disease-free survival, both with statistically significant differences.

**Conclusion:**

The expression levels of lncRNAs are closely associated with the prognosis of AML patients and may serve as important indicators for monitoring prognosis in the future. However, further high-quality studies are needed to validate these findings.

## 1 Background

Acute myeloid leukemia (AML) is one of the most common malignant diseases in the hematological system, characterized by the malignant proliferation of hematopoietic stem cells. It predominantly affects adults and is estimated to be the 11th most common cancer globally (Döhner et al., 2015; Sasaki et al., 2021). Despite significant advancements in AML treatment, including intensive chemotherapy, allogeneic stem cell transplantation, and targeted therapy, which have gradually become the primary treatment modalities and have achieved certain outcomes, the prognosis of AML remains poor, with a 5-year survival rate of less than 25% (Liu, 2021; Rm et al., 2019). Consequently, improving the prognosis of AML has become a key focus for many researchers. Since the European LeukemiaNet (ELN) introduced a method for predicting AML patient prognosis based on cellular and genetic abnormalities in 2017, numerous scholars have been dedicated to identifying indicators for monitoring AML prognosis (Liu, 2021; Döhner et al., 2017). In recent years, long non-coding RNA (lncRNA), an RNA molecule longer than 200 nucleotides that does not code for proteins, has gained attention due to its abnormal expression in various human diseases. LncRNAs regulate gene expression through mechanisms such as epigenetic modification, transcription, and post-transcriptional regulation, and play a role in the development of multiple diseases (Lu et al., 2024). Multiple studies have demonstrated that lncRNAs play a significant role in the pathogenesis of AML (7–31) and show substantial value in diagnosis, prognosis monitoring, and targeted therapy. However, the relationship between lncRNA dysregulation and AML prognosis remains controversial. Therefore, this study aims to use meta-analysis to investigate the evidence-based relationship between lncRNA dysregulation and AML prognosis, providing references for risk stratification and treatment of AML.

## 2 Materials and methods

### 2.1 Document retrieval

Search the Chinese and English databases, including PubMed, Embase, Cochrane Library, China National Knowledge Infrastructure (CNKI), Wanfang Data Knowledge Service Platform, VIP Database, and China Biomedical Literature Service (SinoMed). Additionally, perform manual searches to obtain relevant literature to the greatest extent possible. The search period ranges from the establishment of the databases to 4 August 2024. The Chinese search terms include “lncRNA,” “acute myeloid leukemia,” and “prognosis.” For the English search terms, a combination of subject headings and free text terms is used, with subject headings including “lncRNA,” “acute myeloid leukemia,” and “prognosis.” An example of the PubMed search strategy is provided in Table 1.

**TABLE 1:** Pubmed search strategy.

Search order	Searches
#1	((((((((((((((((((((((((((((“RNA, Long Noncoding” [Mesh]) OR (Noncoding RNA, Long)) OR (RNA, Long Untranslated)) OR (Long Untranslated RNA)) OR (Untranslated RNA, Long)) OR (lncRNA)) OR (Long ncRNA)) OR (ncRNA, Long)) OR (Long ncRNAs)) OR (ncRNAs, Long)) OR (Long Non-Coding RNA)) OR (Long Non Coding RNA)) OR (Non-Coding RNA, Long)) OR (RNA, Long Non-Coding)) OR (RNA, Long Non-Coding)) OR (Long Non-Protein-Coding RNA)) OR (Long Non Protein Coding RNA)) OR (Non-Protein-Coding RNA, Long)) OR (RNA, Long Non-Protein-Coding)) OR (Long Noncoding RNA)) OR (RNA, Long Non-Translated)) OR (Long Non-Translated RNA)) OR (Non-Translated RNA, Long)) OR (RNA, Long Non Translated)) OR (Long Intergenic Non-Protein Coding RNA)) OR (Long Intergenic Non Protein Coding RNA)) OR (LincRNA)) OR (LINC RNA)) OR (LincRNAs)
#2	(((((“Prognosis”[Mesh]) OR (Prognoses)) OR (Prognostic Factors)) OR (Prognostic Factor)) OR (Factor, Prognostic)) OR (Factors, Prognostic)
#3	(((((((((((((((((((((((((((((((((((((((((((((((“Leukemia, Myeloid, Acute”[Mesh]) OR (Leukemia, Acute Myeloid)) OR (Acute Myelogenous Leukemia)) OR (Acute Myelogenous Leukemias)) OR (Leukemias, Acute Myelogenous)) OR (Myelogenous Leukemias, Acute)) OR (Leukemia, Acute Myelogenous)) OR (Myelocytic Leukemia, Acute)) OR (Acute Myelocytic Leukemia)) OR (Acute Myelocytic Leukemias)) OR (Leukemia, Acute Myelocytic)) OR (Leukemias, Acute Myelocytic)) OR (Myelocytic Leukemias, Acute)) OR (Leukemia, Myelocytic, Acute)) OR (Leukemia, Myeloblastic, Acute)) OR (Myeloblastic Leukemia, Acute)) OR (Acute Myeloblastic Leukemia)) OR (Acute Myeloblastic Leukemias)) OR (Leukemia, Acute Myeloblastic)) OR (Leukemias, Acute Myeloblastic)) OR (Myeloblastic Leukemias, Acute)) OR (Leukemia, Myelogenous, Acute)) OR (Myelogenous Leukemia, Acute)) OR (ANLL)) OR (Nonlymphocytic Leukemia, Acute)) OR (Acute Nonlymphocytic Leukemia)) OR (Acute Nonlymphocytic Leukemias)) OR (Leukemia, Acute Nonlymphocytic)) OR (Leukemias, Acute Nonlymphocytic)) OR (Nonlymphocytic Leukemias, Acute)) OR (Leukemia, Nonlymphocytic, Acute)) OR (Leukemia, Nonlymphoblastic, Acute)) OR (Nonlymphoblastic Leukemia, Acute)) OR (Acute Nonlymphoblastic Leukemia)) OR (Leukemia, Acute Nonlymphoblastic)) OR (Leukemias, Acute Nonlymphoblastic)) OR (Nonlymphoblastic Leukemias, Acute)) OR (Acute Myeloid Leukemia)) OR (Acute Myeloid Leukemias)) OR (Leukemias, Acute Myeloid)) OR (Myeloid Leukemias, Acute)) OR (Myeloid Leukemia, Acute)) OR (Myeloid Leukemia, Acute, M1)) OR (Leukemia, Myeloid, Acute, M1)) OR (Acute Myeloid Leukemia without Maturation)) OR (Leukemia, Myeloid, Acute, M2)) OR (Acute Myeloid Leukemia with Maturation)) OR (Myeloid Leukemia, Acute, M2)
#4	#1 OR #2 OR #3

### 2.2 Criteria for inclusion and exclusion

#### 2.2.1 Inclusion criteria

1. Study Subjects: Healthy individuals or patients with benign diseases as the control group, and patients with acute myeloid leukemia (AML) as the case group (age of study subjects ≥14 years).
2. Research Content: Studies examining the prognostic correlation of lncRNA in tissue specimens and AML.
3. Research Type: Cohort studies.
4. Outcome Indicators: Studies must report overall survival (OS), event-free survival (EFS), relapse-free survival (RFS), and disease-free survival (DFS). Hazard ratios (HR) and their 95% confidence intervals (CI) must be either directly extractable or indirectly extractable from survival curves, along with the type, expression level, and cutoff value of lncRNA.

#### 2.2.2 Exclusion criteria

1. Studies published repeatedly or in different languages.
2. Review articles, conference papers, dissertations, and other non-primary research articles.
3. Studies with incomplete data or where the full text is unavailable.
4. Research content not related to the topic.
5. Studies where outcome indicators do not meet the specified criteria or cannot be extracted.

### 2.3 Screening of literature and extraction of data

The retrieved literature was independently screened and included by two researchers in strict adherence to the inclusion and exclusion criteria. In the event of disagreement, a third researcher would arbitrate the decision. The data to be extracted included: ① authors, publication year ② study region ③ sample size ④ detection method ⑤ type, expression level, and cutoff value of lncRNA ⑥ source of samples ⑦ disease type and stage ⑧ follow-up time ⑨ outcome indicators ⑩ source of HR extraction, etc.

### 2.4 Evaluation of literature quality

For the included cohort studies, the authors will employ the Newcastle-Ottawa Scale (NOS) to assess the quality of the literature. Studies scoring ≥6 on the NOS will be considered high-quality and deemed suitable for inclusion in the meta-analysis.

### 2.5 Statistical approaches

Meta-analysis was conducted using Stata16SE software, and the results were presented in a forest plot. The relationship between lncRNA and the prognosis of AML patients was assessed using the combined effect size (HR) and its corresponding 95% *CI* values. To evaluate the heterogeneity of the studies, the authors used the Q test and the *I2* statistic. If the test results indicated *I2* < 50% and *P* > 0.05, it suggested low heterogeneity, and a fixed-effects model was applied; if the test results showed *I2* > 50% and *P* ≤ 0.05, it indicated substantial heterogeneity, and a random-effects model was used. Sensitivity analysis was performed for studies with significant heterogeneity, and subgroup analysis was conducted to explore the sources of heterogeneity. When the number of studies included in the outcome measure exceeded 10, Begg’s test and Egger’s test were employed to assess publication bias.

## 3 Results

### 3.1 Results of literature screening

**FIGURE 1:**
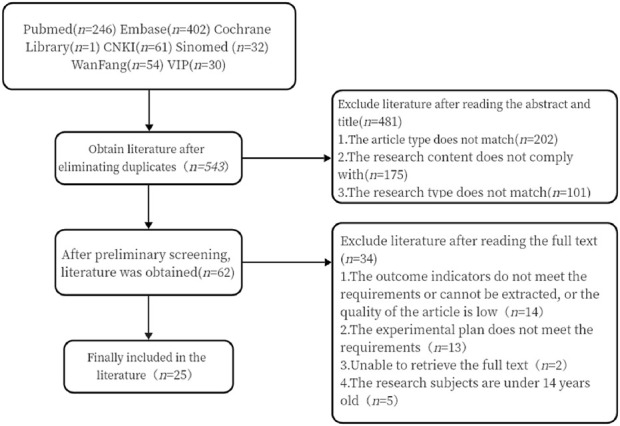
Literature screening process diagram

After conducting the search in strict accordance with the inclusion and exclusion criteria, 826 Chinese and English articles were retrieved. After eliminating 283 duplicates, 62 articles were initially selected based on the abstracts and titles. Finally, 25 articles were included after reviewing the full texts (Figure 1).

### 3.2 The basic characteristics of the included literature and the quality evaluation of NOS

A total of 25 articles were included, with the research period spanning from 2015 to 2024. The research regions included China (20 articles), Egypt (3 articles), and Iran (2 articles). A total of 2,372 patients diagnosed with AML were involved in these studies. The studies examined 22 types of lncRNAs, and the detection method used in all studies was qRT-PCR. The NOS scores of all studies were ≥6 points, indicating their suitability for inclusion in the meta-analysis. The specific details are presented in Table 2.

**TABLE 2:** Basic characteristics of included literature.

Author	Year	Region	Type	Sample size	Test method	lncRNA types and expression levels	Cut-off	Source of Materials	Disease type and staging	Outcome indicators	Source of HR and 95% CI extraction	NOS score
Ma and Li (2024)	2024	CN	R	47	qRT-PCR	HEIH↑	mean	BM	initial diagnosis,non-M3	OS	KM curve	6
Lu et al. (2022)	2022	CN	R	73	qRT-PCR	FOXD3-AS1↑	ROC	BM	initial diagnosis,non-M3	OS	KM curve	6
Zou and Wang (2024)	2024	CN	R	120	qRT-PCR	DANCR↑ SNHG7↑	mean	PB	not mention	OS	KM curve	6
He et al. (2022)	2022	CN	R	87	qRT-PCR	MALAT-1↑	mean	PB	initial diagnosis	OS	KM curve	6
Wang et al. (2023a)	2023	CN	R	88	qRT-PCR	XIST↑	mean	PB	not mention	OS	Text	6
Shan et al. (2022)	2022	CN	R	116	qRT-PCR	HOXA-AS2↑	mean	PB	initial diagnosis	OS	Text	6
Wang et al. (2023c)	2023	CN	R	106	qRT-PCR	CRNDE↑	mean	PB	initial diagnosis,non-M3	OS	Text	6
Huang et al. (2017)	2017	CN	R	56	qRT-PCR	MALAT-1↑	median	BM	initial diagnosis (M5)	OS	Text	7
Abdelrahman et al. (2023)	2023	EG	R	80	qRT-PCR	ZEB2-AS1↑	ROC	BM	initial diagnosis	DFS	Text	7
Gamaleldin et al. (2021)	2021	EG	P	100	qRT-PCR	ANRIL↑ SNHG14↑	median	BM	initial diagnosis,non-M3	OS/RFS	Text	7
Shi et al. (2019)	2019	CN	R	42	qRT-PCR	ZEB2-AS1↑	upper quartile	BM	initial diagnosis	OS/DFS	Text	7
Qu et al. (2020)	2020	CN	R	108	qRT-PCR	HOXA-AS2↑	median	BM	not mention	OS/RFS	Text	7
Wu et al. (2015)	2015	CN	R	85	qRT-PCR	HOTAIR↑	median	BM/PB	not mention	OS	Text	7
Yang et al. (2018)	2018	CN	R	119	qRT-PCR	PANDAR↑	ROC	BM	initial diagnosis	OS	Text	7
Pashaiefar et al. (2018)	2018	IR	R	64	qRT-PCR	IRAIN↓	median	BM	initial diagnosis,non-M3	OS/RFS	Text	7
Luo et al. (2018)	2018	CN	P	73	qRT-PCR	TUG1↑	median	BM	Recurrent or refractory	OS	Text	7
Wang et al. (2018)	2018	CN	P	186	qRT-PCR	TUG1↑	median	BM	initial diagnosis,non-M3	OS/EFS	Text	7
Li and Sun (2018)	2018	CN	R	194	qRT-PCR	SNHG5↑	median	BM/PB	initial diagnosis	OS	Text	7
Wang et al. (2023b)	2023	CN	R	72	qRT-PCR	RBM5-AS1↑	mean	BM	AML	OS	Text	7
Zhou et al. (2020)	2020	CN	R	76	qRT-PCR	NEAT1↑	mean	PB	AML	OS	Text	7
Jia et al. (2018)	2018	CN	R	48	qRT-PCR	KCNQ1OT1↑	mean	PB	AML	OS	Text	6
Bai et al. (2024)	2024	CN	R	50	qRT-PCR	UCA1↑	median	BM	AML	OS	Text	7
Hola et al. (2021)	2021	EG	R	200	qRT-PCR	CRNDE↑	median	PB	initial diagnosis	OS/EFS	Text	7
He et al. (2020)	2020	CN	R	122	qRT-PCR	MEG3↓	median	BM	initial diagnosis,non-M3	OS/EFS	Text	7
Masoud Eslami et al. (2021)	2022	IR	R	60	qRT-PCR	NORAD↑	median	BM	initial diagnosis,non-M3	OS/RFS	Text	7

Note: R: Retrospective study; P: Prospective research; BM: Bone marrow blood; PB: Peripheral blood; OS: Overall survival rate; RFS: recurrence free survival rate; DFS: Event free survival rate; EFS: Disease free survival rate.

### 3.3 The outcomes of the meta-analysis

#### 3.3.1 The correlation between aberrant lncRNA expression and overall survival (univariate analysis) in AML patients

**FIGURE 2:**
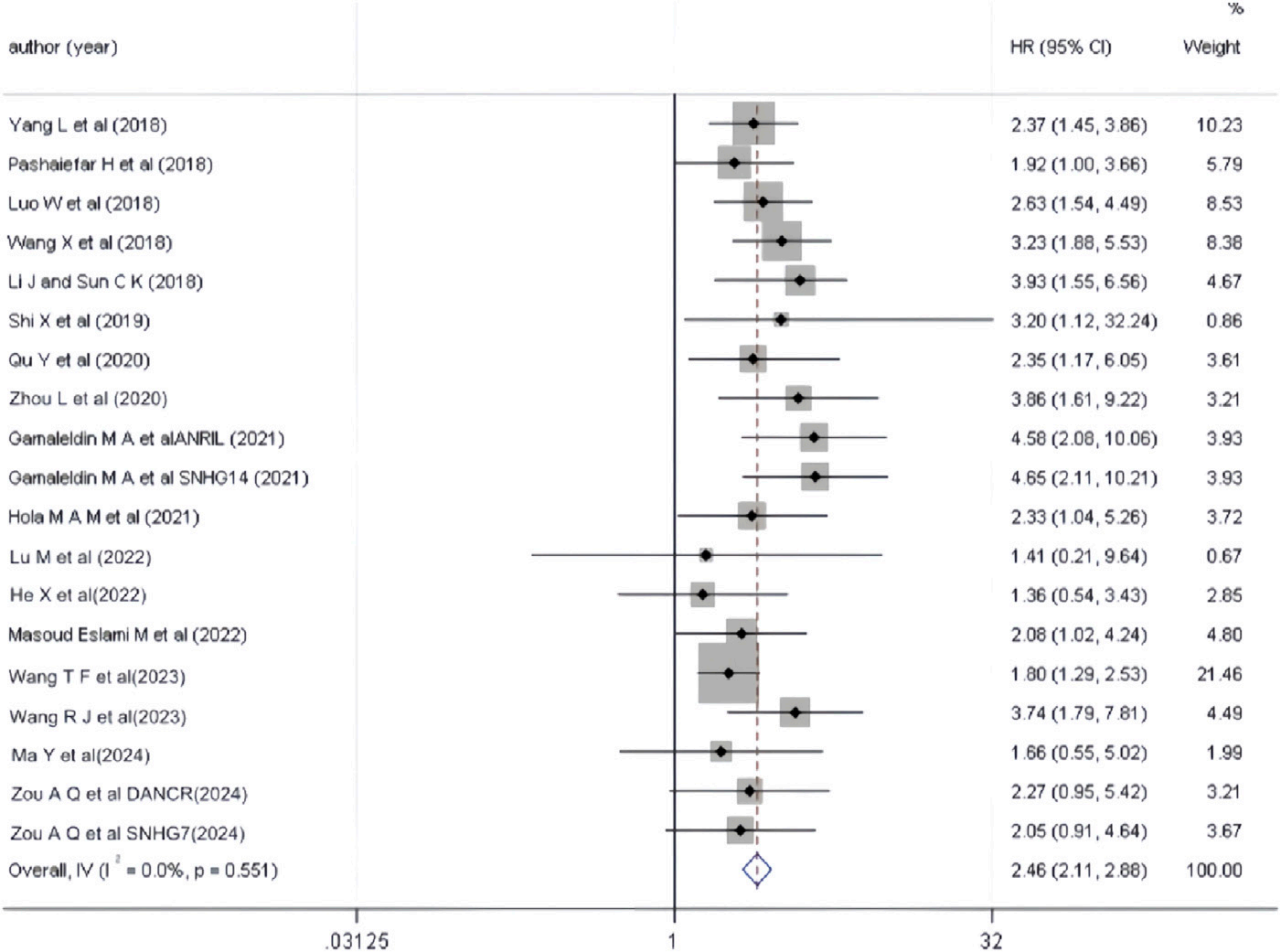
Forest plot of the relationship between abnormal expression of lncRNA and OS (univariate) in AML patients.

17 studies have utilized univariate analysis to investigate the relationship between aberrant lncRNA expression and overall survival (OS) in AML patients. Heterogeneity analysis (*I* ^*2*^ = 0.0%, *P* > 0.05) indicated no significant heterogeneity, leading to the use of a fixed-effects model for the meta-analysis. The results (Figure 2, *HR* = 2.46, 95% *CI* 2.11–2.88, *P* < 0.001) demonstrated that the aberrant expression of lncRNAs, including high expression of DANCR, SNHG7, HEIH, FOXD3-AS1, MALAT-1, PANDAR, TUG1, ZEB2-AS1, NEAT1, ANRIL, SNHG14, SNHG5, CRNDE, NORAD, RBM5-AS1, and low expression of IRAIN, was significantly associated with reduced OS in AML patients, with a statistically significant difference.

#### 3.3.2 The correlation between aberrant lncRNA expression and overall survival (multivariate) in AML patients

**FIGURE 3:**
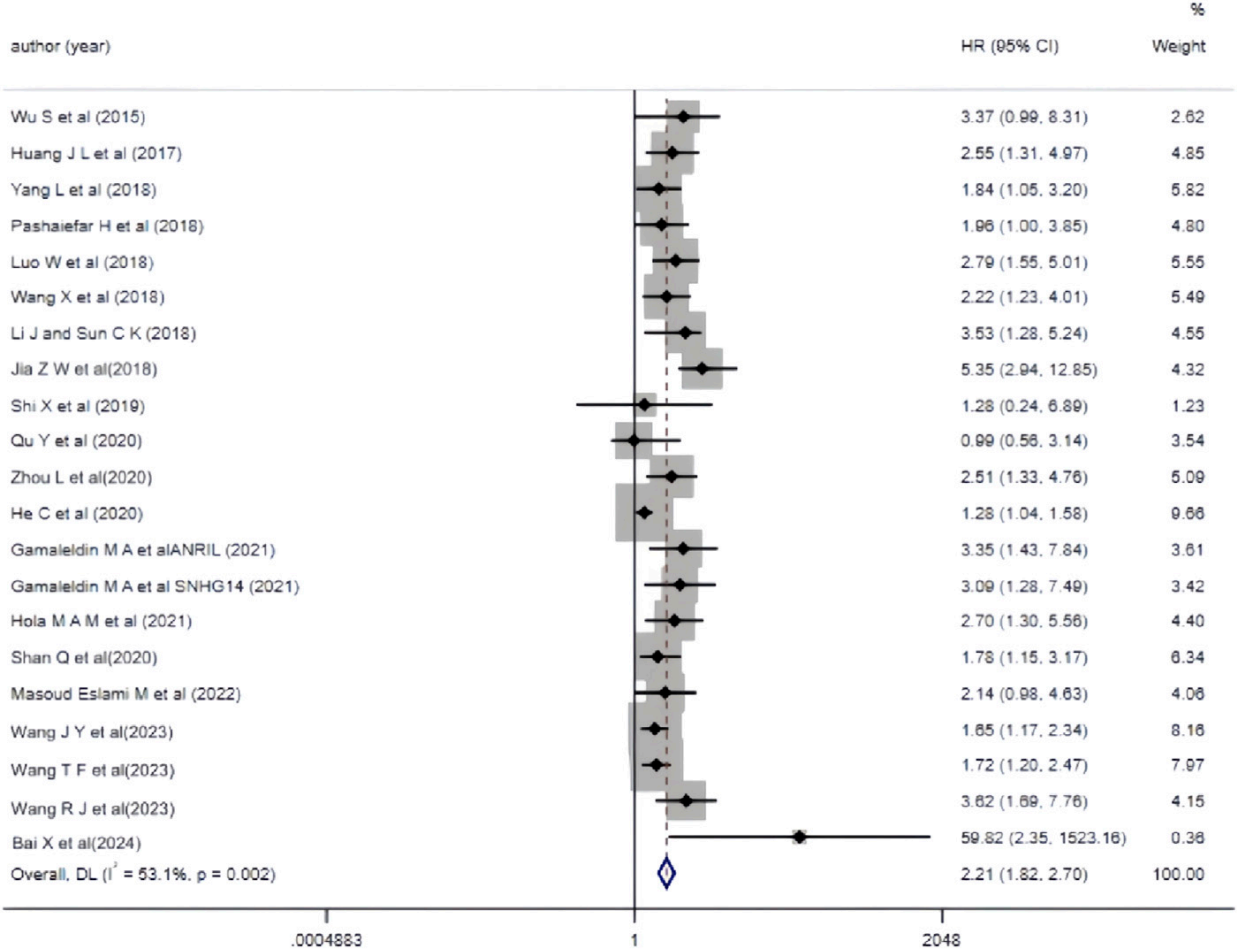
Forest plot of the relationship between abnormal expression of lncRNA and OS (multifactorial) in AML patients.

The 19 included studies utilized multivariate analysis to investigate the relationship between aberrant lncRNA expression and overall survival (OS) in AML patients. Heterogeneity analysis revealed significant heterogeneity (*I* ^*2*^ = 53.1%, *P* < 0.05), prompting the use of a random-effects model for the meta-analysis. The meta-analysis results (Figure 3) showed that AML patients with aberrant lncRNA expression (high expression of XIST, HOXA-AS2, CRNDE, MALAT-1, ANRIL, SNHG14, ZEB2-AS1, HOTAIR, PANDAR, TUG1, SNHG5, RBM5-AS1, NEAT1, KCNQ1OT1, UCA1, CRNDE, NORAD, etc., and low expression of MEG3, IRAIN) had a shorter OS, indicating a poor prognosis. The difference was statistically significant (*HR* = 2.21, 95% *CI* 1.82–2.70, *P* < 0.001). Additionally, we summarized all the factors included in the multivariate analysis of each study with OS as the outcome variable to facilitate the exploration of potential sources of heterogeneity or error in the meta-analysis (Table 3).

**TABLE 3:** Summary of factors in multiple factor analysis of different studies.

Types of lncRNAs	Factors	Types of lncRNAs	Factors	Types of lncRNAs	Factors
NEAT1	risk stratification	HOXA-AS2 (Qu Y et al.)	WBC	HOXA-AS2 (Shan Q et al.)	WBC
	FAB classification		MRD positivity		Prognostic risk stratification
	WBC	ZEB2-AS1	Medical Research Council (favorable/intermediate vs. adverse)		miR-124-3p relative expression level
	Hb		European Leukemia Net (favorable/intermediate vs. adverse)		Age
	PLT	TUG1(Wang X F et al.)	Age	KCNQ1OT1	Sex
	CLAG treatment (vs. FLAG treatment)		Male		Subtype
	Age		WBC		NCCN grouping
	Male (vs. female)		Risk stratification		NCCN grouping
	Relapsed disease (vs. refractory)		WBC		Extramedullary infiltration
	Secondary disease (vs. primary)		HGB	XIST	WBC
TUG1(Luo W et al.)	Poorer risk stratification	HOTAIR	PLT		miR-196b relative expression level
	Higher ECOG performance score		Blasts in BM		FAB classification
	BM blasts		Complete remission	RBM5-AS1	WBC
	CR at first induction		WBC		Proportion of bone marrow primitive cells
	Previous allo-HSCT		AGE		WBC
	Second or higher salvage therapy (vs. first)		Risk classification	CRNDE (Wang T F et al.)	PLT
MEG3	Poorer risk stratification	PANDAR	FLT3-ITD mutation		NCCN grouping
MALAT-1	WBC		CEBPA mutation		Extramedullary infiltration
SNHG5	Cytogenetics		c-KIT mutation		miR-384 relative expression level
	FAB classification		N/K-RAS mutation		Proportion of bone marrow primitive cells
SNHG14 and ANRIL	FLT3/ITD		IDH1/2 mutation		Risk stratification
	CR vs. NCR		U2AF1 mutation		FAB classification
NORAD	Age		Sex	NEAT1	WBC
	Cytogenetic risk group		age		Hb
	FLT3		WBC		PLT
	NPM1		Hb		
CRNDE	BM blasts		PLT		
	FLT3-ITD		BM blasts		
	NPM1	UCA1	Karyotype (normal vs. abnormal)		
			Gene mutation (existent vs. non-existent)		
			Prognostic risk stratification		

Furthermore, we conducted subgroup analyses based on study region, sample size (number of cases), source of material, and disease type to explore the sources of heterogeneity. The results (Table 4) indicated that each subgroup analysis consistently demonstrated that aberrant lncRNA expression was associated with a reduced OS.

**TABLE 4:** Subgroup analysis results.

subgroup	Number of studies	sample size	HR (95%CI)	*P*	effect model	heterogeneity	
						*I* ^*2*^	*P*
Region							
CN	16	1,541	2.157 (1.709–2.722)	<0.001	random effect	60.60%	0.001
Non-CN	5	524	2.510 (1.778–3.543)	<0.001	random effect	0%	0.848
Sample size							
≥100	10	1,351	1.928 (1.502–2.474)	<0.001	random effect	52.50%	0.026
<100	11	714	2.558 (1.941–3.370)	<0.001	random effect	35.60%	0.114
Source of Materials							
PB	6	634	2.152 (1.600–2.993)	<0.001	random effect	50%	0.075
BM	13	1,152	2.132 (1.608–2.828)	<0.001	random effect	55%	0.009
PB or BM	2	279	3.482 (1.934–6.268)	<0.001	random effect	0%	0.943
Disease type							
AML	13	1,271	2.409 (1.846–3.144)	<0.001	random effect	45.60%	0.037
AML (non-M3)	7	738	1.869 (1.418–2.462)	<0.001	random effect	47.60%	0.075
AML (M5)	1	56	2.551 (1.309–4.971)	0.006	random effect	·	·

#### 3.3.3 The correlation between aberrant lncRNA expression and DFS, EFS, and RFS (multivariate variables) in AML patients

**FIGURE 4:**
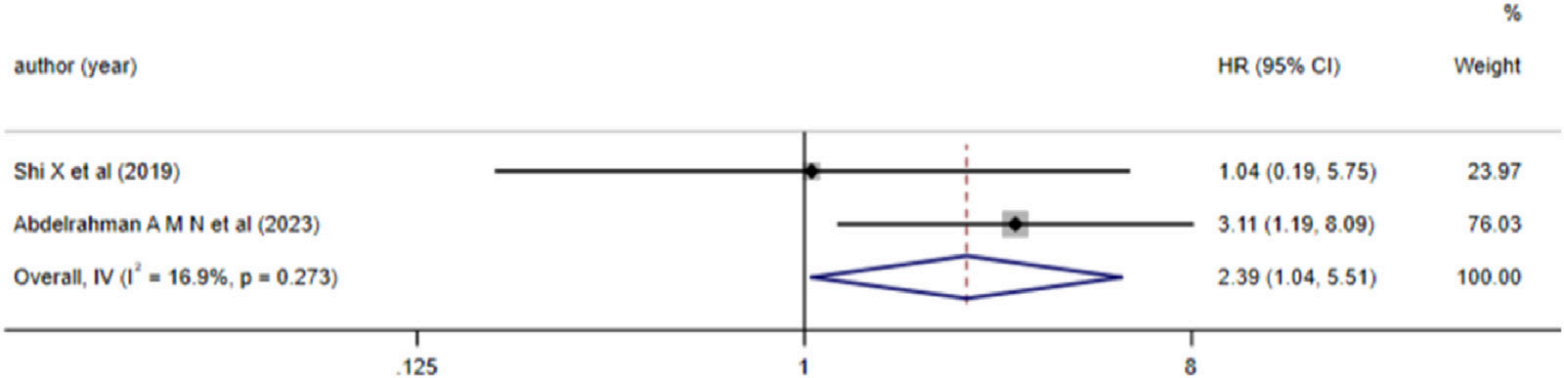
Forest plot of the relationship between lncRNA expression levels and DFS in AML patients.

2 studies reported the correlation between aberrant lncRNA expression and DFS in AML patients. Heterogeneity analysis indicated *I* ^*2*^ = 16.9% and *P* > 0.05, leading to the use of a fixed-effects model for analysis. The results (Figure 4) demonstrated that high expression of lncRNA ZEB2-AS1 was associated with a decreased DFS in AML patients (*HR* = 2.390, 95%*CI* 1.037–5.507, *P* = 0.041), with a statistically significant difference.

**FIGURE 5:**
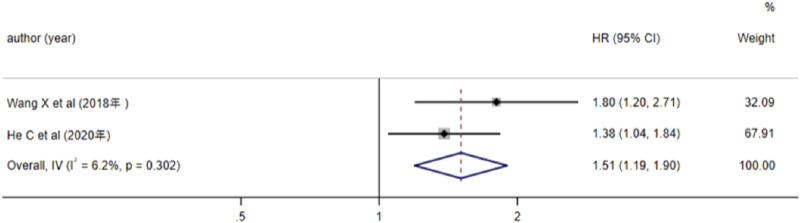
Forest plot of the relationship between lncRNA expression levels and EFS in AML patients.

3 studies analyzed the relationship between lncRNA and AML prognosis, with EFS as the outcome measure. Since the results of Hola et al. (*P* < 0.05) were not statistically significant, we excluded this study and included the remaining two in the meta-analysis. Heterogeneity analysis revealed *I2* = 6.2% and *P* > 0.05, thus a fixed-effects model was adopted. The results (Figure 5) indicated that high expression of lncRNA TUG1 and low expression of lncRNA MEG3 were associated with a reduced EFS in AML patients (*HR* = 1.51, 95%*CI* 1.19–1.90, *P* = 0.001), with a statistically significant difference.

**FIGURE 6:**
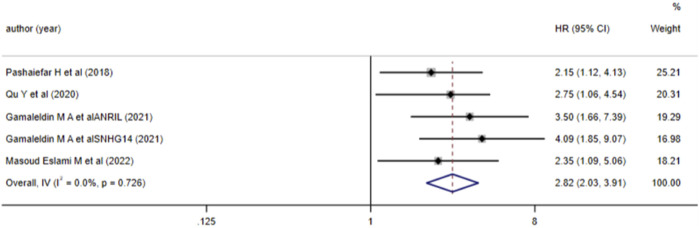
Forest plot of the relationship between lncRNA expression levels and RFS in AML patients.

5 studies investigated the relationship between aberrant lncRNA expression and RFS in AML patients. Heterogeneity testing (*I* ^*2*^ = 0.0%, *P* > 0.05) led to the use of a fixed-effects model for meta-analysis. The results (Figure 6) showed that aberrant expression of lncRNA (high expression of NORAD, ANRIL, SNHG14, HOXA-AS2, etc., and low expression of IRAIN) was associated with a reduced RFS in AML patients (*HR* = 2.82, 95%*CI* 2.03–3.91, *P* < 0.001), with a statistically significant difference.

#### 3.3.4 The relationship between the high expression of lncRNA HOXA-AS2, CRNDE, ZEB2-AS1, TUG1, and MALAT-1 and the prognosis of AML patients

**FIGURE 7:**
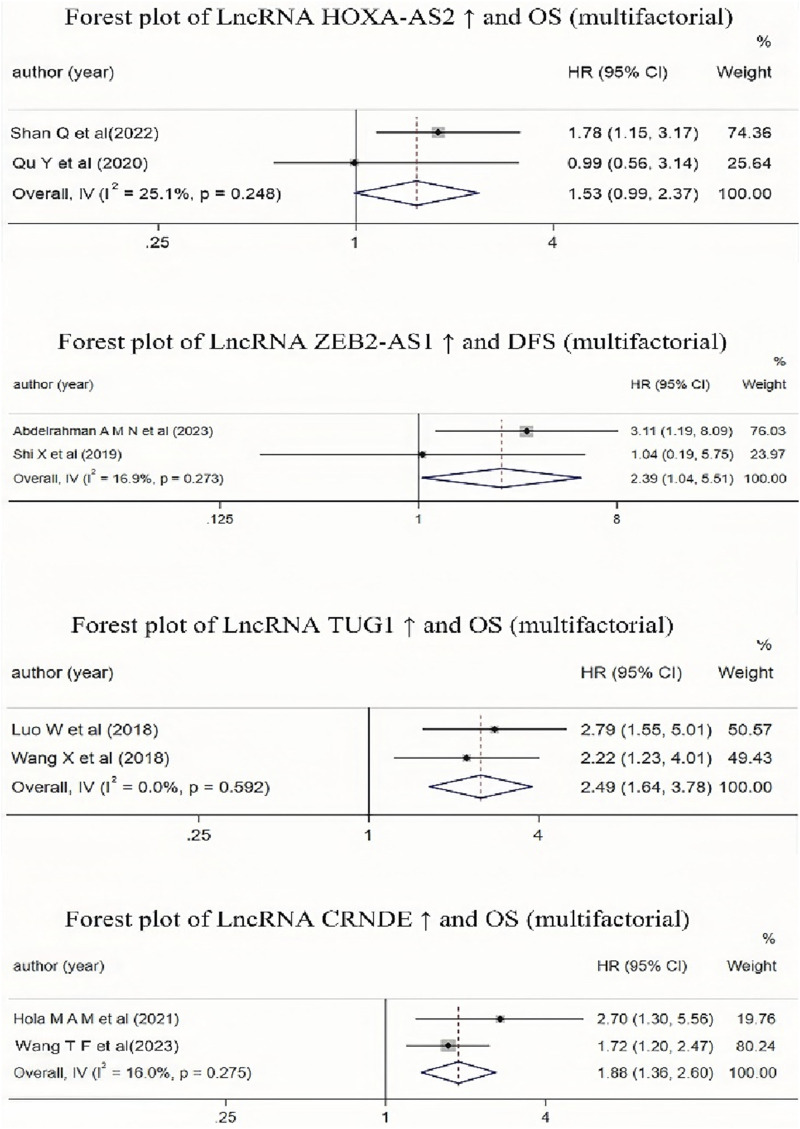
Forest plot collection of different lncRNAs and OS (multifactor) or lncRNAs and DFS(multifactor).

It is evident from Table 2 that multiple studies included in the literature have investigated the impact of high expression of lncRNA HOXA-AS2, CRNDE, ZEB2-AS1, TUG1, and MALAT-1 on the prognosis of AML patients. Due to the different result types of the studies on lncRNA MALAT-1, these results could not be combined. Therefore, we conducted a Meta-analysis to examine the relationship between lncRNA ZEB2-AS1 and DFS, and the relationships between the other three lncRNAs (CRNDE, TUG1, and HOXA-AS2) and OS. The results (as shown in Figure 7) indicated that high expression of lncRNA CRNDE and TUG1 was associated with a reduced OS in AML patients, and high expression of lncRNA ZEB2-AS1 was associated with a decreased DFS in AML patients, with statistically significant differences. However, high expression of lncRNA HOXA-AS2 was associated with a reduced OS in AML patients, but this association was not statistically significant.

#### 3.3.5 Publication bias and sensitivity analysis

We conducted a publication bias assessment using the included literature with OS (single factor or multiple factors) as the outcome indicator. Begg’s test *(P* = 0.944) and Egger’s test (*P* = 0.346) indicated no publication bias in the literature with OS (single factor) as the outcome indicator. However, the literature with OS (multiple factors) as the outcome indicator showed a certain degree of publication bias (Begg’s test: *P =* 0.020, Egger’s test: *P* < 0.001).

**FIGURE 8:**
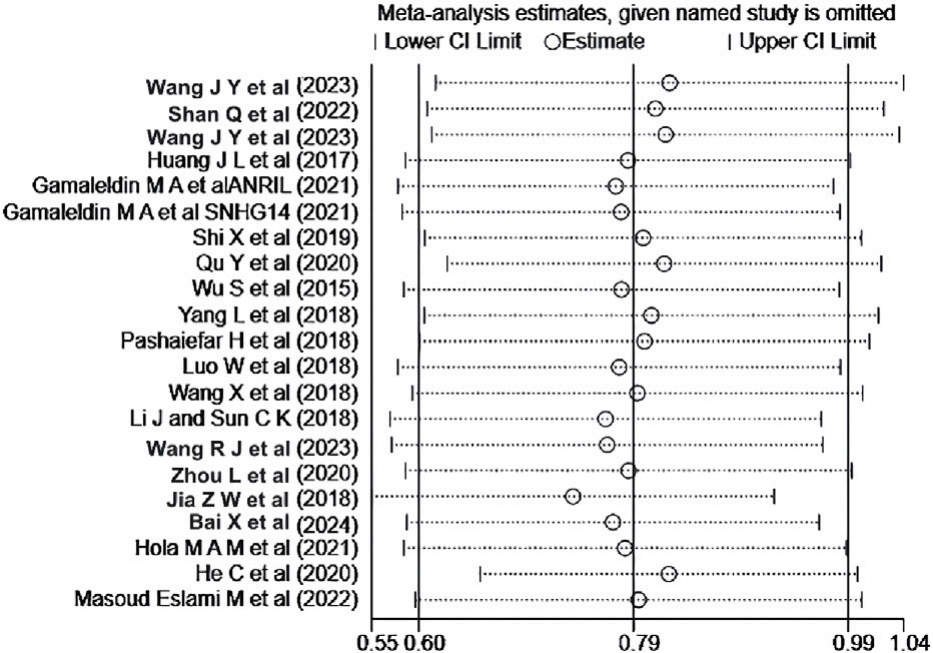
Sensitivity analysis of the relationship between abnormal expression of lncRNA and OS (multifactorial) in AML patients.

A sensitivity analysis was performed for the OS of AML patients in the multiple-factor analysis. The results (Figure 8) indicated that the studies by Jia et al. (2018) and He et al. (2020) significantly influenced the heterogeneity of the Meta-analysis. After excluding these studies and re-analyzing the heterogeneity, the heterogeneity analysis result decreased from *I* ^*2*^ = 53% to *I* ^*2*^ = 9.3%, with *P* = 0.345, *HR* = 2.147, 95%*CI* (1.839 - 2.507), *P* < 0.001. No significant change was observed in the combined effect size after exclusion, indicating good stability of the results.

## 4 Discussion

AML is a hematological disorder characterized by high malignancy and rapid disease progression, with complex etiological factors and significant heterogeneity. Currently, the primary treatment modalities for AML include intensive chemotherapy, targeted therapy, and hematopoietic stem cell transplantation, which have significantly improved the 5-year survival rate of AML patients. However, some patients still face challenges such as poor prognosis, high drug resistance, and high recurrence rates. Therefore, effective prognosis monitoring and the identification of therapeutic targets have become key research priorities for many scholars.

Initially, lncRNA was discovered and considered as “noise” in gene transcription due to its non-coding nature for proteins. However, with advancements in life sciences and gene sequencing technologies, researchers have gradually recognized the critical role of lncRNA in the regulation of biological processes. The types of lncRNA are extremely diverse, and their mechanisms of action are highly complex, and our understanding of them remains limited. Currently, researchers believe that lncRNA plays significant roles in epigenetics, cell apoptosis, differentiation, and other processes, and its involvement has been identified in the development of many diseases. Mumbach et al. published an article in “Nature Methods” using HiCHIRP technology to elucidate the mechanism by which lncRNA regulates chromatin structure and gene expression, demonstrating the significant application value of lncRNA in the field of life sciences (Mumbach et al., 2019). Additionally, with the application of third-generation sequencing technology, a technical foundation has been established for the research and application of lncRNA in clinical medicine. Therefore, exploring the role of lncRNA in the occurrence and development of AML is a highly significant approach for identifying new therapeutic targets and effective prognosis monitoring indicators for AML patients. We employed a meta-analysis method to incorporate studies examining the relationship between lncRNA and the prognosis of AML patients, providing evidence-based medical evidence for the clinical application of lncRNA and offering a valuable reference for scholars researching the relationship between lncRNA and AML.

Our research findings indicate that the abnormal expression of lncRNA has a negative impact on the overall survival (OS), disease-free survival (DFS), relapse-free survival (RFS), and event-free survival (EFS) of AML patients, thereby reducing their prognosis. This is consistent with the previous meta-analysis results of Lin Hongli et al., Shi et al., and Priya (Lin et al., 2022; Shi Jie et al., 2020; Priya et al., 2024). With the in-depth research on lncRNA and AML by scholars, we designed a relatively rigorous meta-analysis protocol, incorporating more recently published studies, and analyzing the relationship between newly discovered lncRNA and the prognosis of AML patients. In terms of the results, there is a certain degree of heterogeneity in studies with OS (using multi-factor analysis) as the outcome indicator. Through subgroup analysis and sensitivity analysis, we believe that the source of heterogeneity may be the different baseline characteristics of the included studies, such as disease types, sample collection methods, and the ethnicity of the study subjects. Additionally, some indicators could not be analyzed due to the small number of studies, non-inclusion in the research protocol, or a large time span of the studies, making it impossible to establish a unified standard, such as the types of lncRNA, expression levels and cut-off values, and AML treatment regimens. We consider that the differences in these baseline characteristics may be the main source of heterogeneity in the included literature, and we hope that more comprehensive and high-quality related studies will emerge in the future. Furthermore, we summarized the various factors in the multi-factor analysis included in the studies and found that the factors explored in each study were not the same. Some studies included comprehensive factors, covering multiple laboratory indicators, and even considered factors such as disease risk stratification and gene mutations, resulting in more reliable outcomes. Some studies only included factors such as age, gender, and white blood cell count, leading to potential positive biases, which may affect the persuasiveness of the resulting evidence-based medical evidence.

For the lncRNAs (HOXA-AS2, CRNDE, ZEB2-AS1, TUG1, MALAT-1) mentioned in multiple articles, we conducted a separate meta-analysis. The results showed that the high expression of lncRNAs (CRNDE, TUG1) reduces the overall survival rate of AML patients, and the high expression of lncRNA ZEB2-AS1 reduces the disease-free survival rate of AML patients. The conclusions were statistically significant, and the heterogeneity of the included literature was relatively small. This suggests that lncRNAs CRNDE, ZEB2-AS1, and TUG1 may have significant potential to become prognostic monitoring indicators for AML patients. However, the number of included studies on lncRNAs (CRNDE, ZEB2-AS1, TUG1) is relatively small, and these three lncRNAs still require further validation to become prognostic risk stratification indicators for AML patients.

**FIGURE 9:**
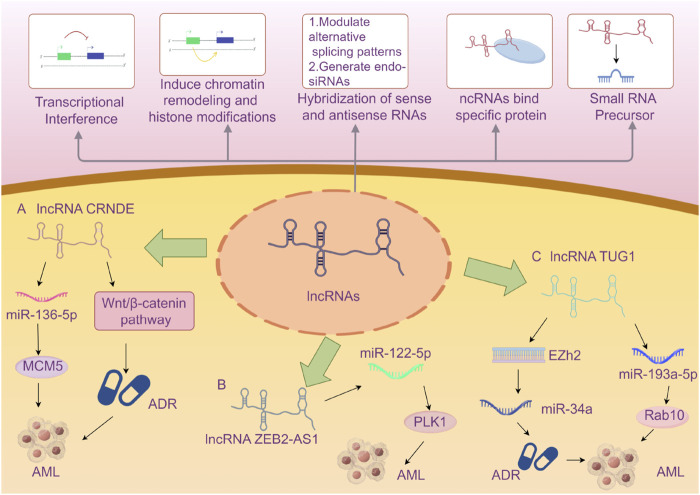
A Brief Introduction to the Functions of lncRNAs (Wilusz et al., 2009) AND The Roles of Three lncRNAs in Acute Myeloid Leukemia. A The lncRNA CRNDE enhances the drug resistance of acute myeloid leukemia (AML) to adriamycin (ADR) by activating the Wnt/β-catenin signaling pathway. CRNDE participates in the CRNDE/miR-136-5p/MCM5 axis to regulate cellular processes and thereby influences the disease progression of AML. B lncRNA ZEB2-AS1 regulates cell proliferation and apoptosis in acute myeloid leukemia via the miR-122-5p/PLK1 axis. C lncRNA TUG1 confers Adriamycin resistance in acute myeloid leukemia by epigenetically suppressing miR-34a expression via EZH2. And it regulates cell viability and apoptosis by modulating the miR-193a-5p/Rab10 axis in acute myeloid leukemia (AML). Note: Extensive research has explored the influence of the aforementioned three specific lncRNAs on AML disease progression via the microRNA/protein axis. However, no definitive consensus has been established to date. The current review may be subject to bias due to potentially incomplete literature inclusion. Future studies should aim to elucidate the mechanisms by which various lncRNAs influence AML progression through more rigorous and compelling experimental designs.

Herein, we summarize the full names, loci, and selected functions of the 22 lncRNAs mentioned in the included literature in tabular form to provide valuable references for readers’ research on the correlation between lncRNAs and AML (Table 5). Due to the limited space in this article, we present an overview of only the 3 lncRNAs that are frequently mentioned in multiple studies (Figure 9).

**TABLE 5:** Summary Table of Multiple lncRNA Information.

lncRNA		Genomic context	Characteristics
HEIH	High expressed in hepatocellular carcinoma	Source of Data:GRCh38 Location:5q35.3, NC_000005.10:180,829,786–180,831,784 Exon count: 1	Research on lncRNA HEIH in hepatocellular carcinoma has been extensive, and it is recognized as an independent prognostic factor. Moreover, it is hypothesized that HEIH may interact with EZH2, potentially modulating the cell cycle (Bill et al., 2019). Currently, there are limited studies on the correlation between lncRNA HEIH and AML. Ma Yue et al. propose that its overexpression may influence the chemoresistance of AML, thereby affecting the prognosis of AML (Ma and Li, 2024).
FOXD3-AS1	Forkhead box D3 antisense 1	Source of Data:GRCh38 Location:1p31.3, NC_000001.11: 63,320,527–63,324,7964 Exon count: 6	Currently, there are limited studies on the correlation between LncRNA FOXD-AS1 and AML. Lu Meng et al. proposed that the abnormal expression of LncRNA FOXD-AS1 may promote the progression of AML by inhibiting the proliferation of Kasumi-1 cells through its suppression (Lu et al., 2022).
DANCR	Differentiation antagonistic non-protein-coding RNA	Source of Data:GRCh38 Location:4q12, NC_000004.12: 52,711,563–52,721,526 Exon count: 5	Studies have shown that the knockdown of lncRNA DANCR can reduce the invasiveness of AML. Kong Wenyan et al. confirmed that lncRNA DANCR regulates the cell cycle of AML cells by targeting miR-656-3p. DANCR has significant potential as a therapeutic target for AML (Bill et al., 2019; Kong et al., 2022).
SNHG7	Small nucleolar RNA host gene 7	Source of Data:GRCh38 Location:9q34.3, NC_000009.12: 136,724,234–136,728,542 Exon count: 5	Currently, the mechanism by which SNHG7 influences AML remains unclear. Shi Jian et al. and Saeed Hassani et al. have confirmed through clinical sample experiments and statistical analyses that SNHG7 is an independent prognostic factor for poor outcomes in AML (Shi et al., 2020b; Hassani et al., 2024).
MALAT-1	Metastasis-associated lung adenocarcinoma transcript 1	Source of Data:GRCh38 Location:11q13.1, NC_000011.10: 65,496,859–65,507,393 Exon count: 3	MALAT-1 is the most abundant and most conserved among lncRNAs and is associated with the metastasis and invasion of multiple cancers. Jin Jing et al. verified through experiments that MALAT-1 regulates the progression of AML by promoting the m6A modification of ZEB1 (Jin et al., 2023).
XIST	X inactive specific transcript	Source of Data:GRCh38 Location:Xq13.2, NC_000023.11: 73,817,449–73,855,919 Exon count: 9	Numerous studies have demonstrated that lncRNA XIST can regulate AML cell proliferation, inhibit cell apoptosis, and consequently influence AML progression. Wang Chong et al. reported that downregulating XIST can decrease MYC expression, thereby reducing drug resistance and promoting apoptosis in AML cells (Wang et al., 2020a; Jiang et al., 2024).
HOXA-AS2	HOXA cluster antisense RNA 2	Source of Data:GRCh38 Location:7p15.2.3, NC_000007.14: 27,121,234–27,129,443 Exon count: 4	Existing studies have demonstrated that the HOXA-AS2-EZH2-LATS2 axis is a critical pathway governing the onset and progression of AML (Feng et al., 2020).
CRNDE	Colorectal neoplasia differently expressed	Source of Data:GRCh38 Location:16q12.2, NC_000016.10: 54,917,860–54,929,887 Exon count: 6	See the following text
ZEB2-AS1	Zinc finger E-box binding homebox 2 antisense 1	Source of Data:GRCh38 Location:2q22.3, NC_000002.12: 144,519,484–144,521,027 Exon count: 4	See the following text
ANRIL	Antisense non-coding RNA in the INK4 locus	Source of Data:GRCh38 Location:9p21.3, NC_000009.12:21,981,455–22,141,476 Exon count: 22	The study by Wang et al. demonstrates that ANRIL is involved in regulating the proliferation and apoptosis of Kasumi-1 cells, which may consequently impact the progression of AML and lead to an unfavorable prognosis (Wang et al., 2024).
SNHG14	Small nucleolar RNA host gene 14	Source of Data:GRCh38 Location:15q11.2, NC_000015.10: 24,764,021–25,479,047 Exon count: 148	Studies on the mechanism of SNHG14 in AML are relatively limited. Wang Xiaoliang et al. demonstrated that silencing SNHG14 to regulate the miR-193b-3p/MCL1 axis decreases the viability of AML cells and promotes their apoptosis (Wang et al., 2021).
HOTAIR	HOX transcript antisense RNA	Source of Data:GRCh38 Location:12q13.13, NC_000012.12: 53,961,047–53,976,217 Exon count: 9	Currently, extensive research has been conducted on HOTAIR. Numerous studies have demonstrated that lncRNA HOTAIR influences the self-renewal of AML stem cells by modulating epigenetic factors or reduces the sensitivity of AML cells to chemotherapy drugs, thereby lowering patient survival rates through multiple pathways (Rajagopal et al., 2020; Gao et al., 2018; Zhou et al., 2021).
PANDAR	Promoter of CDKN1A Antisense DNA Damage Activated RNA	Source of Data:GRCh38 Location:6p21.2, NC_000006.12: 36,673,469–36,675,276 Exon count: 1	Reports on LncRNA PANDAR are more commonly found in various solid tumors and less frequently in hematological neoplasms. Yang Lan et al. assessed the expression levels of PANDAR in AML patients and concluded that its overexpression is associated with poor prognosis in AML (Yang et al., 2018).
IRAIN	IGF1 receptor antisense imprinted noncoding RNA	Source of Data:GRCh38 Location:15q26.3, NC_000015.10: 98,645,369–98,651,279 Exon count: 1	IRAIN is an antisense long non-coding RNA of IGF1R and is related to the intramolecular loop structure between the DNA promoter and enhancer of IGF1R. (Liang et al., 2020) IRAIN is involved in the interaction with chromatin DNA. Studies have shown that low expression of IRAIN is closely associated with a reduced complete remission rate in AML patients (Pashaiefar et al., 2018; Sun et al., 2014).
TUG1	Taurine-upregulation gene 1	Source of Data:GRCh38 Location:22q12.2, NC_000022.11: 30,968,251–30,980,407 Exon count: 4	See the following text
SNHG5	Small nucleolar RNA host gene 5	Source of Data:GRCh38 Location:6q14.3, NC_000006.12: 85,676,833–85,678,905 Exon count: 6	A bioinformatics and meta-analysis study indicates that the overexpression of SNHG5 is associated with poor prognosis in various cancers, including colorectal cancer and AML (Pashirzad and Sahebkar, 2024). Furthermore, multiple studies have experimentally confirmed that SNHG5 is involved in cell proliferation, apoptosis, and chemotherapy resistance in AML (Li and Sun, 2018; Priya et al., 2024)
RBM5-AS1	Long non-coding RNA binding motif protein 5-antisense strand 1	Source of Data:GRCh38 Location:3p21.31, NC_000003.12: 50,099,463–50,101,126 Exon count: 1	Currently, there are limited studies on the correlation between RBM5-AS1 and AML. One study demonstrated that RBM5-AS1 is highly expressed in the bone marrow of AML patients and may influence cell proliferation, migration, and invasion by modulating the Wnt/β-catenin pathway (Wang et al., 2023b).
NEAT1	Nuclear paraspeckle assembly transcript 1	Source of Data:GRCh38 Location:11q13.1, NC_000011.10: 65,420,523–65,447,813 Exon count: 1	At present, numerous studies have demonstrated that NEAT1 controls the proliferation and apoptosis of AML cells by regulating gene expression via miRNA (Yan et al., 2021; Rostami et al., 2022).
KCNQ1OT1	KCNQ1 opposite strand/antisense transcript 1	Source of Data:GRCh38 Location:11p15.5, NC_000011.10: 2,599,160–2,709,160 Exon count: 1	KCNQ1OT1 accelerates the progression of AML and enhances chemoresistance via the miR-193a-3p/TSpan3 axis (Sun et al., 2020).
UCA1	Urothelial carcinoma-associated 1	Source of Data:GRCh38 Location:19p13.12, NC_000019.10: 15,828,208–15,837,058 Exon count: 3	UCA1 is capable of inhibiting the proliferation of AML cells and promoting cell apoptosis. This effect has been validated in studies involving both adult and pediatric AML patients. Furthermore, Xiao et al. compared the expression levels of lncRNA UCA1 in exosomes from AML patients and those who achieved complete remission after chemotherapy, concluding that exosomal lncRNA UCA1 could serve as a crucial biomarker for the diagnosis and treatment monitoring of AML, providing a novel approach for more convenient monitoring of AML prognosis and development (Xiao et al., 2022; Liang et al., 2020; Wilson et al., 2023).
MEG3	Maternally expressed gene 3	Source of Data:GRCh38 Location:14q32.2, NC_000014.9: 100,822,615–100,864,517 Exon count: 13	Current research suggests that MEG3 may influence the onset and progression of AML by modulating the expression of p53 (Lyu et al., 2017).
NORAD	Noncoding RNA activated by DNA damage	Source of Data:GRCh38 Location:20q11.23, NC_000020.11: 36,045,083–36,051,493 Exon count: 1	Currently, there is relatively limited research on the mechanisms of NORAD in AML. Masoud Eslami et al. demonstrated that high NORAD expression is a critical factor associated with poor prognosis in non-M3 AML patients, based on comparisons of NORAD expression levels in clinical samples from non-M3 AML patients and healthy individuals (Masoud Eslami et al., 2021).

LncRNA CRNDE (Colorectal Neoplasia Differently Expressed) was initially identified with aberrant expression in colorectal neoplasms and is located on the reverse strand of chromosome 16 (Thierry-Mieg and Thierry-Mieg, 2006). Subsequent in-depth studies have revealed that lncRNA CRNDE exhibits abnormal expression in various solid tumors, such as glioma, hepatocellular carcinoma, and lung cancer, as well as in hematological malignancies. It has been demonstrated that lncRNA CRNDE holds significant value in the diagnosis, treatment, and prognosis of certain tumors (Wang T. et al., 2023; Hola et al., 2021; Ma et al., 2022; Ma et al., 2019). Numerous studies have indicated that lncRNA CRNDE may be involved in the occurrence and development of cancer through its regulatory interactions with microRNAs and proteins (Hor et al., 2023; Xie et al., 2018). Ma et al. demonstrated that knocking out CRNDE in acute promyelocytic leukemia (APL) mice could induce APL cell differentiation, inhibit proliferation, and extend the survival time of APL mice (Ma et al., 2020). Liu et al. proved that CRNDE participates in the CRNDE/miR-136-5p/MCM5 axis to regulate cellular processes and thereby influences the disease progression of AML (Liu et al., 2021). From an evidence-based medicine perspective, our research has shown that the risk of poor prognosis in AML patients with high expression of lncRNA CRNDE is 1.88 times higher than that in patients with low expression. CRNDE may become an important indicator of poor prognosis in AML in the future, but further exploration is still necessary.

LncRNA ZEB2-AS1 (zinc finger E-box binding homeobox 2 antisense 1) is an antisense long non-coding RNA that undergoes antisense transcription during protein coding and is located on the long arm of chromosome 2 at band 2q3. Studies have indicated that lncRNA ZEB2-AS1 shows increased expression in various cancers (Mahboobeh et al., 2020). Guan et al. verified through bioinformatics analysis and mouse experiments that lncRNA ZEB2-AS1 regulates the proliferation and apoptosis of AML cells via the miR-122-5p/PLK1 axis (Guan et al., 2020). The study by Abdelrahman et al., which we included, concluded that lncRNA ZEB2-AS1 can be regarded as a predictor of poor prognosis for AML in Egyptians (Abdelrahman et al., 2023). We combined this study with the one by Shi et al. for meta-analysis and concluded that high expression of lncRNA ZEB2-AS1 reduces the disease-free survival (DFS) of AML patients. ZEB2-AS1 is expected to become an important indicator for prognosis monitoring of AML patients in the future, but more comprehensive studies are still required for validation.

LncRNA TUG1 (taurine-upregulated gene 1) is a 7.1-kb long non-coding RNA located on human chromosome 22. Multiple studies have confirmed that the abnormal expression of lncRNA TUG1 is closely related to the disease progression of various conditions, particularly cardiovascular diseases (Chang and Su, 2024; Liu B. et al., 2024; Liu Y. et al., 2024). It may influence the occurrence and progression of diseases by regulating gene expression or interacting with specific proteins as a competitive endogenous RNA (ceRNA) (Li et al., 2016). Sun et al. reconstructed the lncRNA-miRNA-mRNA network and identified three lncRNAs (XIST, TUG1, GABPB1-AS1) as potential key lncRNAs for cytogenetically normal AML from a bioinformatics perspective, demonstrating that lncRNA TUG1 might be related to the prognosis of AML. This article was the first to propose that lncRNA GABPB1-AS1 might be an important biomarker related to the prognosis of AML (Sun et al., 2022). Other studies have suggested that TUG1 might be associated with doxorubicin resistance in AML, thereby affecting the prognosis of AML (Li et al., 2019). We combined two related studies on TUG1 and concluded that the risk of reduced overall survival (OS) in AML patients with high expression of lncRNA TUG1 is 2.49 times higher than that in patients with low expression. TUG1 may become a new tool for risk stratification of AML in the future, but further research on its mechanism of action is still awaited.

From the existing studies, it is evident that no single lncRNA can directly predict the prognosis of AML or provide unambiguous evidence for risk stratification of AML. Similar to the complex pathogenesis of AML, the regulatory mechanisms of lncRNAs involved in the disease are also intricate, likely influencing the disease through the joint participation of one or more lncRNAs. Therefore, this highlights the need to consider the variations in the expression of multiple lncRNAs in the disease progression when researching the prognostic risk stratification of lncRNAs for AML. Scores should be assigned based on the significance of their roles in the process, thereby establishing a comprehensive scoring system for evaluating the prognosis of AML using lncRNAs. Additionally, the current highly efficient testing techniques can provide substantial assistance for the diagnosis, treatment, and prognosis detection of AML.

Limitations of this study: ① In some studies, hazard ratios (HRs) were not provided directly; instead, we extracted survival data from Kaplan-Meier (KM) curves, which may introduce certain errors. ② The types, expression levels, and cutoff values of lncRNAs included in this study varied, potentially impacting the reliability of the results. ③ The majority of the included studies originated from China, which may affect the generalizability of the findings. ④ The majority of the included studies reported positive results, suggesting the presence of publication bias that could not be entirely eliminated.

In conclusion, the abnormal expression of lncRNAs is associated with poor prognosis in AML, demonstrating considerable potential to serve as prognostic monitoring indicators and tools for risk stratification of AML. Specifically, the evidence from evidence-based medicine indicates a clear correlation between the high expression of lncRNAs (CRNDE, ZEB2-AS1, TUG1) and poor prognosis in AML. Future research can further validate these findings through various approaches, including bioinformatics, basic experiments, and clinical studies. However, whether lncRNAs can become reliable prognostic risk stratification tools for AML remains to be determined. More large-scale, multi-center, high-quality studies are needed to explore the relationships between the expression levels of different lncRNAs and the baseline characteristics of AML patients, such as different subtypes of AML, the effectiveness of different treatment regimens, and patient age.
